# Ultra-processed foods intake and sex hormone levels among children and adolescents aged 6–19 years: a cross-sectional study

**DOI:** 10.3389/fnut.2024.1451481

**Published:** 2024-09-06

**Authors:** Hao Zhao, Wei Gui, Shangtao Liu, Fangyu Zhao, Wenyan Fan, Fangyuan Jing, Chuan Sun

**Affiliations:** ^1^Department of Preventive Medicine, School of Basic Medical Sciences, Jiujiang University, Jiujiang, China; ^2^Jiangxi Provincial Key Laboratory of Cell Precision Therapy, School of Basic Medical Sciences, Jiujiang University, Jiujiang, China; ^3^Department of Pediatric, The Affiliated Hospital of Jiujiang University, Jiujiang, China; ^4^Department of Molecular Mechanisms of Chronic Diseases, Shulan International Medical College, Zhejiang Shuren University, Hangzhou, China; ^5^Zhejiang Key Laboratory of Geriatrics and Geriatrics Institute of Zhejiang Province, Zhejiang Hospital, Hangzhou, China

**Keywords:** ultra-processed foods, sex hormones, child, adolescent, cross-sectional

## Abstract

**Background:**

Sex hormones are crucial for the development of children and adolescents. The increasing consumption of ultra-processed foods (UPFs) among children and adolescents in the United States (US) has raised concerns about their potential impact on health, including hormonal balance.

**Methods:**

Data from 3,354 participants aged 6–19 years from the NHANES 2013–2016 were analyzed. UPF intake was categorized using the NOVA food classification system, and the percentage of total daily energy intake from UPFs was calculated. The serum levels of total testosterone (TT), sex hormone-binding globulin (SHBG), and estradiol (E2) were measured. The free androgen index (FAI) and TT/E2 ratio were calculated to estimate bioavailable testosterone levels and the balance between androgens and estrogens, respectively. Multiple linear regression models, adjusted for potential confounders, estimated the associations.

**Results:**

Our results showed that higher intake of UPFs was marginally associated with decreased serum SHBG levels (quartile (Q) 2 vs. Q1: *β* = −5.3, 95% confidence interval (CI): −17.0, 8.1%; Q3 vs. Q1: *β* = −14.6, 95%CI: −25.1, −2.5%; Q4 vs. Q1: *β* = −9.0, 95%CI: −20.3, 3.8%; *P* trend = 0.081), and significantly associated with increased serum FAI in female adolescents (Q2 vs. Q1: *β* = 3.2, 95%CI: −3.3, 9.7; Q3 vs. Q1: *β* = 7.6, 95%CI: −0.7, 16.0; Q4 vs. Q1: *β* = 9.5, 95%CI: 1.5, 17.6; *P* trend = 0.019). Additionally, UPF intake showed a marginally positive association with increased serum SHBG levels (*P* trend = 0.057) in male children and FAI (*P* trend = 0.150) in male adolescents, respectively. Similar results were observed when participants were stratified by puberty status, except for the association between UPF intake and SHBG in male children. However, there were no associations between UPF consumption and TT, E2, or the TT/E2 ratio, both in males and females.

**Conclusion:**

Higher UPF consumption is associated with increased FAI in adolescents, particularly in girls, indicating higher bioavailable testosterone levels. Future studies should validate these findings with direct free testosterone measurements and more precise dietary intake assessments.

## Introduction

UPFs, as defined by the NOVA classification system, are industrial formulations made primarily from processed food substances. These substances contain little or no whole food and typically include preservatives, colorings, flavorings, and other additives designed to enhance flavor, extend shelf life, and improve texture (1). UPFs are typically energy-dense, high in calories, added sugar, unhealthy fats, and salt while being low in dietary fiber, protein, vitamins, and minerals.

In a recent global analysis of trends, the volume sales of UPFs were highest in North America and Australasia (2). Particularly concerning is the increase in the proportion of energy intake from UPFs among U.S. youths, which increased from 61.4 to 67.0% between 1999 and 2018 (3). This trend is alarming given the strong evidence that increased UPF intake among children and adolescents has been correlated with overweight, obesity, physical inactivity, and periodontal diseases (4). Recent studies have also found that UPF consumption is associated with increased exposure to endocrine disruptors (EDCs), such as phthalates, bisphenols, and perfluorooctane sulfonate (5–7). These compounds are known to interfere with the body’s hormonal balance (8, 9). Additionally, obesity among children and adolescents can adversely affect sex hormone homeostasis (10).

Steroid sex hormones are crucial for physical growth and development, as well as the maintenance of metabolic homeostasis, bone health, and muscle function in children and adolescents (11). Previous studies have suggested that sex hormone levels may be disrupted by dietary intake (12–16). Most of these studies have focused on specific nutrients (12–14, 16) or specific dietary patterns, such as the Mediterranean diet (12), vegetarian diet (12), and pro-inflammatory diet (15). Additionally, sex hormones in childhood and adolescence may respond differently to external environmental disruptions (17). However, to the best of our knowledge, the impact of UPFs on sex hormone homeostasis remains under-explored among children and adolescents.

In the NHANES survey, dietary intake data were collected by trained interviewers using the United States Department of Agriculture’s (USDA) Automated Multiple-Pass Method (AMPM). The AMPM was designed to provide an efficient and accurate means of collecting intakes for large-scale national surveys. The data from the NHANES have shown higher exposure to UPFs among the U.S. general population, including children and adolescents. The NHANES 2013–2016 cycles include serum TT, SHBG, and E2 measurements in participants ≥6 years of age. Because of existing concerns about UPF intake on sex hormone levels, the current study evaluated the relationship of UPF intake with serum sex hormone levels among male children (ages 6–11 years) and adolescents (ages 12–19 years) in a representative U.S. population sample.

## Methods

### Study population

The population of this cross-sectional study was derived from the National Health and Nutrition Examination Survey (NHANES) 2013–2016 cycle. The NHANES utilized a sophisticated, stratified, multistage probability survey design to obtain a nationally representative sample of the civilian, non-institutionalized U.S. population (18). The 2013–2016 cycles included 20,146 participants from 60 different survey locations. The data were collected via household interviews, phone interviews, and physical examinations. The NHANES protocol was approved by the National Center for Health Statistics (NCHS) Research Ethics Review Board. Informed consent was obtained from all NHANES participants (18).

**Figure 1:**
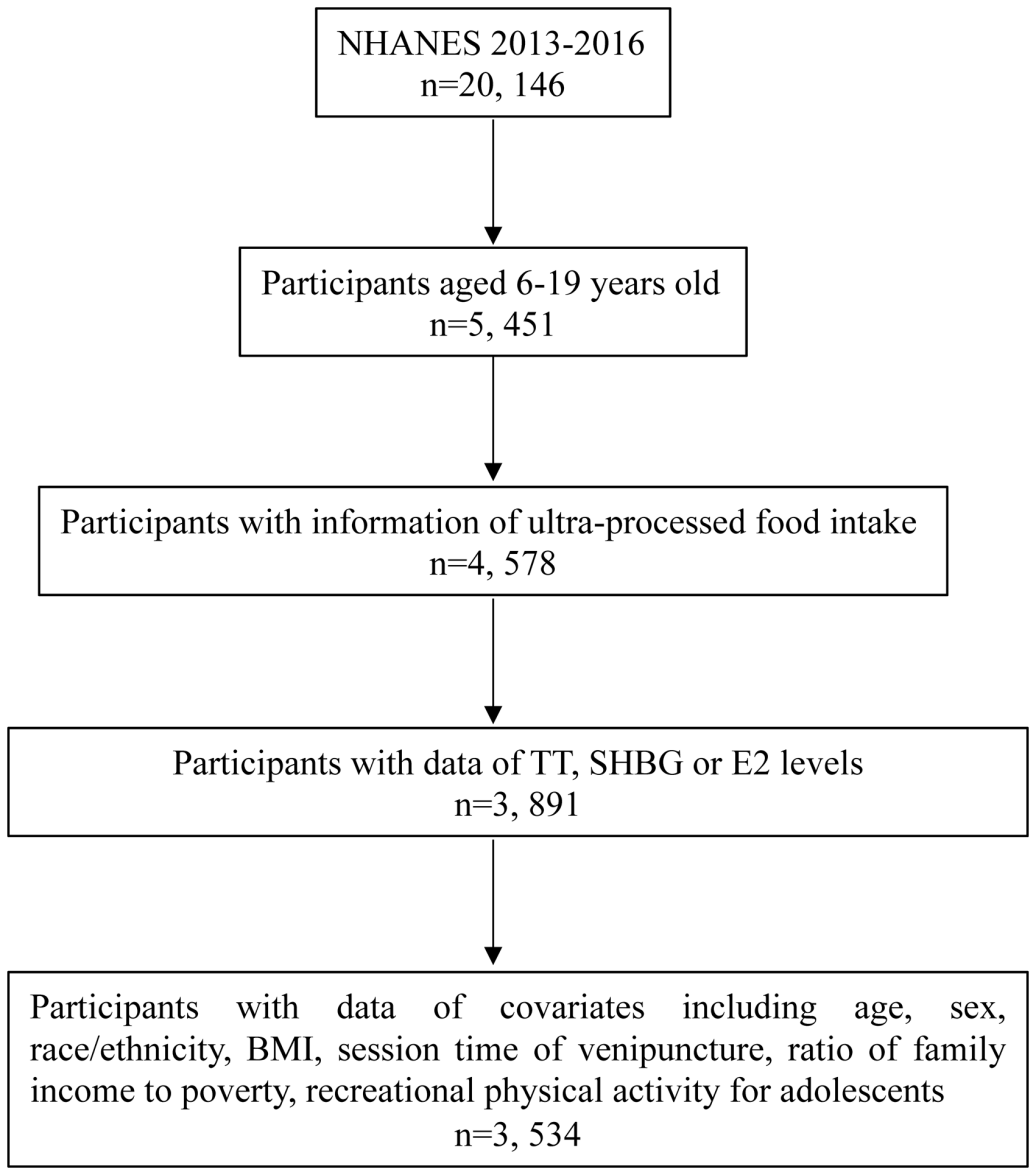
Flow chart of participants selection. TT, total testosterone; SHBG, sex hormone-binding globulin; E2, estradiol; BMI, body mass index.

Of these, 5,451 participants were aged 6–19 years. Furthermore, participants were excluded if they lacked food intake information (*n* = 873) or data on serum TT, SHBG, or E2 levels (*n* = 687). Additionally, participants without data of covariates including age, sex, race/ethnicity, body mass index (BMI), the session time of venipuncture, the ratio of family income to poverty, and recreational physical activity for adolescents were excluded (*n* = 357). Finally, a total of 3,534 participants were included in the data analysis (Figure 1).

### Dietary assessment and estimated UPF intake

Dietary intake was assessed using two non-consecutive 24-h dietary recall interviews by trained interviewers using the USDA AMPM (19). The AMPM was designed to provide an efficient and accurate means of collecting intakes for large-scale national surveys. The first interview-administered dietary recall was collected in person at the mobile examination center (MEC), whereas the second was collected via telephone 3–10 days later. This study included participants who reliably completed at least one of the dietary recalls. Dietary intake was reported as the average intake from both 24-h recalls when data from 2 days were available and as data from day 1 otherwise.

In the dietary interviews, participants provided information on the types and amounts of food and beverages they consumed the previous day. The food items reported by participants were categorized into four mutually exclusive groups based on the NOVA food classification system: (1) unprocessed or minimally processed foods, (2) processed culinary ingredients, (3) processed foods, and (4) UPFs. The methods for food classification were described elsewhere (20). Briefly, the foods were assigned to each of the four groups based on the variables “main food description,” “additional food description,” and “SR code description” from the NHANES 24-h recall datasets. Classification could be modified according to the variables “Combination Food Type” and “Source of Food.” For instance, most foods described as “Frozen meals” or “Lunchables,” as well as some items described as consumed in “Restaurant fast food/pizza” or acquired at a “Vending machine,” were classified as UPFs. For items considered handmade recipes, the NOVA classification was applied to each underlying ingredient [standard reference (SR) codes] as previously described (20). Based on the above classification, specific food items, identified by their respective food codes and SR codes, have been classified into UPFs and 18 subgroups (Supplementary Table S1).

The percentage of total daily energy intake from UPFs was calculated by the food code energy values provided by What We Eat in America (WWEIA), the NHANES, and the Food and Nutrient Database for Dietary Studies (FNDDSs). For handmade recipes, we calculated the underlying ingredient (SR Codes) energy values using data from both the FNDDS and USDA National Nutrient Database for Standard Reference (20). Two researchers independently reviewed the classifications, resolving any discrepancies by consensus. The primary exposure measure in this study was the mean dietary contribution of UPFs to total energy intake.

### Measurement of sex hormone levels

Serum E2 and TT levels were quantified using isotope dilution liquid chromatography–tandem mass spectrometry (ID-LC–MS/MS), and SHBG was analyzed based on its reaction with immuno-antibodies and chemo-luminescence measurements. The limit of detection (LOD) for TT, E2, and SHBG was established as 0.75 ng/dL, 2.994 pg./mL, and 0.800 nmol/L, respectively. For values below the LOD, calculations were adjusted to LOD/√2 to accommodate for lower detection capabilities. To assess the bioavailable testosterone, the FAI was calculated using the formula [(TT × 100)/SHBG]. Additionally, the ratio of TT to E2 (TT/E2) was computed to indirectly assess the circulating free testosterone levels. These calculations offer insights into the hormonal balance and potential alterations in sex hormone levels (21). The detection rates of serum E2 were 8.7 and 48.5% for male and female children, respectively (Supplementary Table S2).

### Covariates

The potential confounders were selected according to previous studies (22, 23). The questionnaires obtained all covariate information, including demographic information such as age (years), gender (male, female), race/ethnicity (Mexican American, other Hispanic, non-Hispanic white, non-Hispanic Black, other race), family poverty-to-income ratio (PIR), and BMI (kg/m2). PIR, calculated by dividing family income by the federal poverty level for family size, was used as an indicator of socioeconomic status. Children and adolescents were classified as underweight, normal weight, overweight, or obese according to age and sex, as defined by NHANE (body measures files^1^).^2^ For current analyses, underweight and normal weight were combined as one category. Furthermore, the time of blood draw (morning, afternoon, and evening) and season of blood collection (1 November to 30 April and 1 May to 31 October) were used as covariates in this analysis. We included this covariate due to the wide variation in sex steroid hormone levels, which can fluctuate diurnally, weekly, and seasonally (24, 25). Information on recreational physical activity was available for adolescents only; participants were asked whether they engaged in regular moderate and/or vigorous recreational activities (categorized as yes or no).

### Statistical analysis

All analyses were performed using the weight from the MEC visit (i.e., MEC weights) to account for the clustered sample design, survey non-response, over-sampling, post-stratification, and sampling error, and to permit generalization to the US population. All analyses were conducted according to the NHANES guidelines (26). SAS 9.4 (SAS Institute Inc., Cary, NC) was used for all statistical analyses. *p*-values were presented at a significance level of <0.05.

Given that sex steroid hormone levels vary significantly by gender and developmental stage, analyses were conducted separately for male children (6–11 years), male adolescents (12–19 years), female children (6–11 years), and female adolescents (12–19 years). Distributions of variables were presented as mean with standard error (SE) for continuous variables and as frequencies and weighted percentages for categorical variables.

The multivariable linear regression model was used to calculate adjusted β coefficients for the associations between the percent of total energy intake from UPFs (categorized into four groups based on quartiles) and TT, SHBG, E2, FAI, and TT/E2. Specifically, the levels of TT, SHBG, and E2 were natural logarithm (Ln) transformed in the linear regression models due to skewed distribution. Subsequently, effect estimates were calculated as [exp (β)-1] × 100%, which indicates the percent change in the outcome variable (ln-transformed) with respect to a one-unit change in the exposure variable. Potential confounders were adjusted for age, gender, race/ethnicity, PIR, BMI, venipuncture session time, 6-month sex hormone examination period, and family income-to-poverty ratio. Recreational physical activity was additionally adjusted for the participants aged 12–19 years. The *P* trend values were calculated by treating the median values of each quartile group as continuous variables.

A series of sensitivity analyses were also performed. Participants were categorized into prepuberty and puberty groups across males and females based on serum sex steroid levels and menarche status. Boys with serum TT levels ≥50 ng/dL and girls with serum E2 ≥ 20 pg./mL were considered to have entered puberty (27). In addition, participants were classified as having entered puberty based on the “age at menarche” reported in the reproductive health questionnaire; those not meeting this criterion were classified as prepuberty. Associations between UPF intake with sex steroid hormone levels were then analyzed separately for prepuberty and puberty stages across both males and females.

Furthermore, UPFs were classified into 18 food items (Supplementary Table S1) (20). The associations of these 18 food items’ percent energy intake with sex steroid hormone levels were analyzed among children (6–11 years) and adolescents (12–19 years) for both males and females.

## Results

Table 1 presents the socio-demographic characteristics of the study population by age and gender. A total of 3,534 participants were included in the study. The mean percent of total energy intake from UPFs among all participants was 66.1% ± 0.5%. No significant differences were observed in UPF intake by race/ethnicity, BMI, or the 6-month time period when the sex hormone examination was performed across gender and age groups (*p* > 0.05). However, significant variations were observed in the session timing of venipuncture, PIR, and recreational physical activity levels among adolescents across different age and gender categories (*p* < 0.05).

**Table 1:** Weighted characteristics for children and adolescent (6–19 years) participants in NHANES 2013–2016 (*n* = 3,534).

Characteristics	Total	Male Children (*n* = 805)	Male Adolescents (*n* = 1,000)	Female Children (*n* = 764)	Female Adolescents (*n* = 965)	*p*-values^c^
Percentage of total energy intake from UPFs ^*^ Mean ± SE or n (%)	66.1 ± 0.5	66.1 ± 0.8	66.3 ± 0.7	65.7 ± 1.1	66.1 ± 0.7	0.968
Q1 (<55.7%)	1,008 (24.9)	218 (23.2)	279 (25.0)	229 (25.5)	282 (25.6)	0.632
Q2 (55.7–<67.3%)	902 (25.1)	221(27.8)	239 (23.1)	198 (24.9)	244 (25.5)	
Q3 (67.3–<78.1%)	825 (25.0)	197 (26.4)	235 (24.0)	180 (25.4)	213 (25.0)	
Q4 (≥78.1%)	799 (25.0)	169 (22.6)	247 (27.9)	157 (24.1)	226 (23.9)	
Race/ethnicity, n (%)						
Mexican American	827 (15.7)	167 (16.1)	220 (14.8)	205 (19.2)	235 (14.6)	0.872
Other Hispanic	405 (7.8)	100 (9.4)	104 (7.2)	81 (7.4)	120 (7.8)	
Non-Hispanic white	993 (54.7)	230 (52.7)	309 (57.7)	203 (50.2)	251 (55.4)	
Non-Hispanic Black	815 (12.8)	205 (13.7)	232 (12.3)	178 (13.3)	200 (12.5)	
Other Race	494 (8.9)	103 (8.1)	135 (8.0)	97 (9.9)	159 (9.7)	
BMI, n (%)^a^						
Normal/underweight	2,113 (61.1)	500 (63.0)	606 (62.0)	457 (64.2)	550 (57.3)	0.324
Overweight	643 (17.9)	136 (16.8)	167 (16.6)	145 (17.1)	195 (20.5)	
Obese	765 (20.9)	169 (20.2)	219 (21.4)	162 (18.8)	215 (22.2)	
Session time of venipuncture, n (%)						
Morning	1,518 (42.9)	295 (35.5)	469 (44.7)	295 (38.8)	459 (48.2)	<0.001
Afternoon	1,288 (36.2)	328 (40.1)	359 (37.4)	292 (37.8)	309 (31.5)	
Evening	728 (20.9)	182 (24.5)	172 (18.0)	177 (23.4)	197 (20.3)	
6-month time period when the sex hormone examination was performed, n (%)						
1 November to 30 April	1738 (45.0)	397 (46.2)	519 (46.9)	352 (41.8)	470 (44.2)	0.404
1 May to 31 October	1796 (55.0)	408 (53.8)	481 (53.1)	412 (58.2)	495 (55.8)	
Ratio of family income to poverty, n (%)						
< 130%	1,544 (31.5)	360 (32.9)	412 (27.9)	361 (37.2)	411 (31.1)	0.023
130–< 350%	1,301 (40.3)	288 (40.4)	374 (40.2)	267 (37.4)	372 (42.1)	
≥ 350%	689 (28.2)	157 (26.7)	214 (32.0)	136 (25.4)	182 (26.8)	
Physical activity, n (%)^b^						
Yes	1,491 (79.5)	−	823 (84.4)	−	668 (74.2)	<0.001
No	447 (20.5)	−	161 (15.6)	−	286 (25.8)	

BMI, body mass index; SE, standard error; UPFs, ultra-processed foods; Q1–Q4 represent the quartile values of percent of total energy intake from UPFs; −, not available. *The classification of the subgroup of ultra-processed foods was based on a previous paper (20).

a Children and adolescents were categorized into underweight, normal weight, overweight, or obese groups based on age and sex, following the criteria outlined by NHANES (http://wwwn.cdc.gov/Nchs/Nhanes/2011-2012/BMX_G.htm).

b Recreational physical activity information was exclusively obtained for adolescents. Participants were queried about their involvement in regular moderate and/or vigorous recreational activities, which were subsequently categorized as either yes or no.

c For continuous variables, the comparison between four subgroups was performed using the ANOVA test. For category variables, the χ^2^ test was used.

Table 2 presents the associations between the percentage of total energy intake from UPFs and sex hormone levels, adjusted for potential confounders. Among male children, those in the highest quartile (Q4) of UPF intake had a 13.1% (95% CI, 0.8, 26.8%, *P* trend = 0.057) increase in serum SHBG levels compared to those in the lowest quartile (Q1). However, UPF intake was not associated with TT or FAI values in male children. In female adolescents, a marginal negative association was observed between UPF intake and serum SHBG levels (Q2 vs. Q1: *β* = −5.3, 95% CI: −17.0, 8.1%; Q3 vs. Q1: *β* = −14.6, 95% CI: −25.1, −2.5%; Q4 vs. Q1: *β* = −9.0, 95% CI: −20.3, 3.8%; *P* trend = 0.081). Additionally, a significant positive dose–response relationship was found between UPF intake and FAI values (Q2 vs. Q1: *β* = 3.2, 95% CI: −3.3, 9.7; Q3 vs. Q1: *β* = 7.6, 95% CI: −0.7, 16.0; Q4 vs. Q1: *β* = 9.5, 95% CI: 1.5, 17.6; *P* trend = 0.019). In male adolescents, significant associations were observed between UPF intake in the second (Q2 vs. Q1: *β* = 126.2, 95% CI: 20.5, 232.0) and third quartiles (Q3 vs. Q1: *β* = 168.0, 95% CI: 21.5, 314.6) and FAI compared to the first quartile, whereas the significant association disappeared among those in the fourth quartile. No significant associations were found between UPF intake and TT in both children and adolescents. Due to the low detection rate of serum E2 among male and female children (Supplementary Table S2), the associations of UPF intake with E2 and the TT/E2 ratio were confined to adolescents. No significant associations were observed between UPF intake and E2 or the TT/E2 ratio. Similar findings were observed in the crude models for these associations mentioned above (Supplementary Table S3).

**Table 2:** Associations between percentage of total energy intake from UPF and sex hormone levels among participants aged 6–19 years old adjusted by potential confounders^*^.

Percentage of total energy intake from UPFs	TT (%)^a^ β (95%CI)	SHBG (%)^a^ β (95%CI)	FAI β (95%CI)	TT/E2 β (95%CI)	E2 (%)^a^ β (95%CI)
**Male children**					
Q1 (<55.7%)	Reference	Reference	Reference	–	–
Q2 (55.7– < 67.3%)	6.1 (−18.8, 38.6)	5.1 (−6.6, 18.4)	−7.6 (−38.8, 23.7)	–	–
Q3 (67.3– < 78.1%)	0.9 (−16.5, 21.8)	4.2 (−6.4, 16.0)	−15.0 (−40.5, 10.5)	–	–
Q4 (≥78.1%)	12.1 (−11.1, 41.3)	**13.1 (0.8, 26.8)**	−3.0 (−36.3, 30.4)	–	–
*P* trend	0.392	0.057	0.695	–	–
**Male adolescent**					
Q1 (<55.7%)	Reference	Reference	Reference	Reference	Reference
Q2 (55.7–<67.3%)	6.8 (−11.4, 28.9)	−2.5 (−11.3,7.1)	**126.2 (20.5, 232.0)**	0.9 (−1.9, 3.7)	1.4 (−11.0, 15.7)
Q3 (67.3–<78.1%)	10.5 (−4.7, 28.0)	−10.5 (−20.9, 1.3)	**168.0 (21.5, 314.6)**	−0.5 (−2.7, 1.8)	10.8 (−1.7, 24.9)
Q4 (≥78.1%)	7.4 (−8.7, 26.4)	−1.1 (−9.8, 8.4)	96.8 (−35.4, 229.0)	0.9 (−0.8, 2.6)	1.0 (−10.5, 14.0)
*P* trend	0.354	0.495	0.150	0.574	0.618
**Female children**					
Q1 (<55.7%)	Reference	Reference	Reference	–	–
Q2 (55.7–<67.3%)	0.0 (−13.6, 15.7)	4.9 (−6.7, 18.0)	0.5 (−3.4, 4.5)	–	–
Q3 (67.3–<78.1%)	7.1 (−4.7, 20.4)	7.0 (−5.8, 21.5)	−1.2 (−6.0, 3.6)	–	–
Q4 (≥78.1%)	2.7 (−10.8, 18.1)	6.0 (−4.7, 17.8)	1.1 (−3.9, 6.0)	–	–
*P* trend	0.506	0.245	0.838	–	–
**Female adolescent**					
Q1 (<55.7%)	Reference	Reference	Reference	Reference	Reference
Q2 (55.7–<67.3%)	−0.3 (−9.5, 9.8)	−5.3 (−17.0, 8.1)	3.2 (−3.3, 9.7)	0.3 (−0.2, 0.8)	−4.2 (−20.5, 15.4)
Q3 (67.3–<78.1%)	−4.8 (−14.0, 5.3)	**−14.6 (−25.1, −2.5)**	7.6 (−0.7, 16.0)	0.1 (−0.3, 0.5)	−6.6 (−27.1, 19.6)
Q4 (≥78.1%)	5.4 (−4.7, 16.5)	−9.0 (−20.3, 3.8)	**9.5 (1.5, 17.6)**	−0.1 (−0.5, 0.2)	6.3 (−14.7, 32.4)
*P* trend	0.520	0.081	**0.019**	0.315	0.734

UPFs, ultra-processed foods; TT, total testosterone; SHBG, sex hormone-binding globulin; E2, estradiol; FAI, free androgen index; CI, confidence interval; Q1–Q4 represent the quartile values of percent of total energy intake from ultra-processed food; −, not available. *The linear regression models were adjusted by age, race/ethnicity, BMI, session time of venipuncture, and 6-month time period when the sex hormone examination was performed and ratio of family income to poverty, while the recreational physical activity was adjusted additionally for the participants aged 12–19 years old.

a Due to TT, SHBG, and E2 levels being ln-transformed, the parameter estimates represent the percent change in outcome variable with respect to one-unit change of UPF intake. The bold values indicated the *P* values <0.05.

Considering the age variation in entering puberty for males and females, participants were classified into prepuberty and puberty groups. The results show patterns similar to those observed in Table 2, except for the association between UPF intake and SHBG in male children (Table 3; Supplementary Table S4).

**Table 3:** Associations between percent of total energy intake from UPF and sex hormone levels among participants aged 6–19 years old grouped by puberty status in NHANES 2013–2016.^*^

Percentage of total energy intake from UPFs	TT (%)^a^ β (95%CI)	SHBG (%)^a^ β (95%CI)	FAI β (95%CI)	TT/E2 β (95%CI)	E2 (%)^a^ β (95%CI)
**Male prepubertal**					
Q1 (<55.7%)	Reference	Reference	Reference	–	–
Q2 (55.7–<67.3%)	−5.7 (−20.2, 11.4)	3.4 (−7.9, 16.1)	1.0 (−3.2, 5.2)	–	–
Q3 (67.3–<78.1%)	2.0 (−12.7, 19.1)	−0.1 (−10.3, 11.3)	2.1 (−1.9, 6.0)	–	–
Q4 (≥78.1%)	10.4 (−4.4, 27.6)	7.0 (−3.6, 18.8)	2.9 (−2.9, 8.7)	–	–
*P* trend	0.130	0.320	0.293	–	–
**Male pubertal**					
Q1 (<55.7%)	Reference	Reference	Reference	Reference	Reference
Q2 (55.7–<67.3%)	7.9 (−2.3, 19.1)	−1.0 (−10.3, 9.3)	**117.1 (16.7, 217.5)**	0.1 (−0.4, 0.7)	−7.3 (−21.4, 9.5)
Q3 (67.3–<78.1%)	4.7 (−4.0, 14.3)	−10.3 (−19.9, 0.5)	**155.0 (20.6, 289.3)**	0.1 (−0.2, 0.4)	−11.8 (−27.8, 7.7)
Q4 (≥78.1%)	8.1 (−0.2, 17.2)	−1.7 (−9.8, 7.1)	106.7 (−12.7, 226)	0.0 (−0.4, 0.4)	2.1 (−19.2, 29.1)
*P* trend	0.121	0.319	0.103	0.952	0.998
**Female prepubertal**					
Q1 (<55.7%)	Reference	Reference	Reference	–	–
Q2 (55.7–<67.3%)	−10.1 (−24.5, 6.9)	5.9 (−9.1, 23.5)	−0.8 (−3.8, 2.2)	–	–
Q3 (67.3–<78.1%)	−1.1 (−12.3, 11.7)	6.3 (−7.0, 21.6)	−1.5 (−4.5, 1.5)	–	–
Q4 (≥78.1%)	−1.2 (−16.8, 17.2)	7.4 (−7.4, 24.6)	0.0 (−2.6, 2.6)	–	–
*P* trend	0.825	0.326	0.844	–	–
**Female pubertal**					
Q1 (<55.7%)	Reference	Reference	Reference	Reference	Reference
Q2 (55.7–<67.3%)	3.9 (−4.4, 13.0)	−5.3 (−15.6, 6.3)	4.9 (−0.9, 10.8)	0.3 (−0.2, 0.7)	−0.5 (−16.6, 18.8)
Q3 (67.3–<78.1%)	−1.2 (−11.0, 9.7)	**−14.4 (−23.7, −3.9)**	**8.8 (1.2, 16.4)**	0.2 (−0.1, 0.6)	−9.3 (−26, 11.1)
Q4 (≥78.1%)	4.2 (−4.9, 14.1)	−6.5 (−17.2, 5.6)	**9.4 (2.2, 16.6)**	0.0 (−0.1, 0.2)	−0.2 (−17, 19.8)
*P* trend	0.638	0.124	**0.015**	0.849	0.762

UPFs, ultra-processed foods; TT, total testosterone; SHBG, sex hormone–binding globulin; E2, estradiol; FAI, free androgen index; CI, confidence interval; Q1–Q4 represent the quartile values of percent of total energy intake from ultra-processed food; −, not available. *The linear regression models were adjusted by age, race/ethnicity, BMI, session time of venipuncture, and 6-month time period when the sex hormone examination was performed and the ratio of family income to poverty, while the recreational physical activity was adjusted additionally for the participants aged 12–19 years old.

a Due to TT, SHBG, and E2 levels being ln-transformed, the parameter estimates represent the percent change in outcome variable with respect to one-unit change of UPF intake. The bold values indicated the *P* values <0.05.

Based on the distribution of the proportion of energy intake from UPF subgroups, the intake of three specific food items (1: bread; 2: salty snacks; 3: sauces, dressings, and gravies) was classified into three groups according to tertile values for further subgroup analysis (Supplementary Table S1). Other food items were categorized into ‘intake’ and ‘non-intake’ groups due to their low frequency of consumption for further analysis.

Supplementary Figures S1–S4 present the associations between UPF subgroups and sex hormone levels across age and sex categories. Some UPF subgroups were negatively or positively associated with specific sex hormone levels. Notably, positive associations were observed between the intake of sandwiches and hamburgers on buns and carbonated soft drinks and FAI among female adolescents (Supplementary Figure S3).

## Discussion

To the best of our knowledge, this is the first study investigating the associations between UPF intake and sex hormone levels in individuals aged 6–19 years old. Our results show that UPF intake is marginally negatively associated with serum SHBG levels and significantly positively associated with serum FAI values in female adolescents or female pubertal individuals. For male adolescents or male pubertal individuals, marginally positive associations were found between UPF intake and FAI. There is limited evidence suggesting that UPF intake is associated with sex hormones in children or prepubertal individuals, except for the associations between UPF intake and serum SHBG in male children. Furthermore, there are no associations between UPF intake and TT, E2, or the TT/E2 ratio among individuals aged 6–19 years old.

However, previous research found that the dietary inflammatory index, which measures the inflammatory potential of a diet based on its components’ impact on inflammatory biomarkers (28), was associated with lower TT and E2 levels in male adolescents (15). Modest reductions in fat intake during puberty were found to be associated with lower E2 levels and higher TT levels during the luteal phase in female adolescents (13). Nevertheless, these modest reductions in fat intake did not alter sex hormone levels in male adolescents (14). In the present study, we did not find significant associations for TT, E2, or the TT/E2 ratio. The differences may be attributed to the methodologies used to classify food intake. However, some subgroup food items of UPFs were positively or negatively associated with TT, E2, and TT/E2 (Supplementary Figures S1–S4). These results suggest that these food items may interact with each other, which could lead to a lack of associations between the sum of UPF intake and TT, E2, and the TT/E2 ratio.

Furthermore, our study indicated that UPF intake was associated with increased FAI values in adolescents, especially in females. The FAI is used to estimate the amount of biologically active or free testosterone in the blood. It is commonly used in both clinical and research settings to assess androgen status, particularly in conditions such as polycystic ovary syndrome (PCOS), hyperandrogenism, and hormonal imbalance in adolescents. The result suggests that high consumption of UPFs could lead to elevated levels of bioavailable testosterone. This could influence pubertal development and hormonal health, potentially increasing the risk of conditions such as PCOS and other androgen-related disorders. A previous study showed that FAI correlated well with free testosterone in females but not in males (29). Additionally, the FAI is not reliable in women when the SHBG concentration is low (30). Thus, future studies with direct free testosterone measurement should be performed to validate the results in adolescents.

Although no studies have explored the associations between UPF intake and free testosterone levels in female adolescents, dietary fat intake was found to be associated with increased FAI in adult females (31) and females with PCOS (32). Additionally, during the production and packaging of UPFs, there may be increased exposure to various chemicals, including phthalates, bisphenols (5, 6), per- and polyfluoroalkyl substances (PFASs) (33), organophosphate (34), acrylamide (35), and micro(nano) plastics (36). Phthalates, bisphenols, PFAS, organophosphates, and micro(nano) plastics are recognized as EDCs, which may affect sex hormone levels (37–40). Acrylamide has been classified as a probable human carcinogen and has also been found to disrupt sex hormone homeostasis among preschool-aged children (41) and youths aged 6–19 years (17). Experimental studies have shown that acrylamide damages hypothalamic gonadotropin-releasing hormone (GnRH) neurons and disrupts the hypothalamic–pituitary–gonadal (HPG) axis in pubertal mice, indicating that adolescence is a particularly vulnerable stage for acrylamide-induced sex hormone disruption (17). These findings somewhat support our results. Thus, exposure to chemicals from UPF packaging and production may be one pathway for UPF-induced sex hormone disruption.

In addition, higher fat and sugar are characteristics of UPFs. Increased consumption of UPFs is associated with obesity in children and adolescents (42). Visceral fat accumulation could reduce SHBG concentrations in both sexes (43), which may explain the association of UPF intake with decreased SHBG and increased FAI among adolescents in the present study.

This study took advantage of the high standards of NHANES quality control on survey methods and data collection to examine the relationship between UPF intake and sex hormone levels in children and adolescents for the first time. Leveraging NHANES, a comprehensive and nationally representative dataset, allows for robust and generalizable findings. However, potential limitations should be taken into consideration. First, even though 24-h recalls are considered the least biased self-report tool available (44) and the standardized methods used by NHANES are known to produce accurate dietary estimates (45, 46), this method still has its limitations when it comes to determining UPF consumption (47). Specifically, the level of food processing (e.g., meal location and brand names) is not always consistently recorded for each food item, and the nutritional information provided may not reflect the most current market offerings (48). Despite these shortcomings, any resulting misclassification in UPF estimates is likely to be non-differential, which means that errors in estimating the dietary contribution of UPFs are expected to be random and thus would likely dilute any true association between UPF consumption and sex hormones toward the null (49). Second, residual confounding could exaggerate the relationship between diet and health outcomes since high UPF consumption often correlates with an overall unhealthy lifestyle. Furthermore, foods reported on assessment days may not accurately represent usual diets, potentially leading to underestimated effects in studies. Third, the cross-sectional nature of the current study precludes us from concluding the causal effect.

## Conclusion

Our study is the first to explore the relationship between UPF intake and sex hormone levels in individuals aged 6–19 years old, using data from the NHANES 2013–2016. The findings indicate that higher UPF intake is associated with increased FAI in female adolescents, suggesting a potential impact on bioavailable testosterone levels. This association was less pronounced in male adolescents and was not observed in prepubertal children, except for a link between UPF intake and SHBG levels in male children.

These results suggest that UPF consumption may influence hormonal health during adolescence, particularly in females. This study highlights the need for further research with longitudinal designs and directs free testosterone measurements to confirm these associations and investigate the mechanisms behind potential hormonal disruptions.

## Data Availability

The datasets presented in this study can be found in online repositories. The names of the repository/repositories and accession number(s) can be found at: https://wwwn.cdc.gov/nchs/nhanes/ContinuousNhanes/Default.aspx?BeginYear=2013 and https://wwwn.cdc.gov/nchs/nhanes/continuousnhanes/default.aspx?BeginYear=2015.

## Footnotes

^1^ https://wwwn.cdc.gov/Nchs/Nhanes/2013-2014/BMX_H.htm

^2^ https://wwwn.cdc.gov/Nchs/Nhanes/2015-2016/BMX_I.htm
