# Supplementary material for: Ultra-processed foods intake and sex hormone levels among children and adolescents aged 6–19 years: a cross-sectional study
Source: Front Nutr. 2024 Sep 6;11:1451481. doi: 10.3389/fnut.2024.1451481 (PMC11412839; doi:10.3389/fnut.2024.1451481)
Supplement: Supplementary file 1 [file Data_Sheet_1.docx]

**Supplementary materials**

**Ultra-processed foods intake and sex hormones levels among children and adolescents aged 6-19 years: a cross-sectional study**

Hao Zhao ^1, 2, *^, Wei Gui ^3^, Shangtao Liu ^1^, Fangyu Zhao ^1^, Wenyan Fan ^1^, Fangyuan Jing ^4, *^, Chuan Sun ^5, *^

^1^ Department of Preventive Medicine, School of Basic Medical Sciences, Jiujiang University, Jiujiang, China.

^2^ Jiangxi Provincial Key Laboratory of Cell Precision Therapy, School of Basic Medical Sciences, Jiujiang University, Jiujiang, China.

^3^ Department of Pediatric, The Affiliated Hospital of Jiujiang University, Jiujiang, China.

^4^ Department of Molecular Mechanisms of Chronic Diseases, Shulan International Medical College, Zhejiang Shuren University, Hangzhou, China.

^5^ Zhejiang Key Laboratory of Geriatrics and Geriatrics Institute of Zhejiang Province, Zhejiang Hospital, Hangzhou, China.

^*^ Corresponding authors at:

**Hao Zhao**, 551 East Road of Qianjin, School of Basic Medical Sciences of Jiujiang University, Jiujiang 332000, China. Email address: [zh1984817@126.com](mailto:zh1984817@126.com).

**Fangyuan Jing**, Shuren street 8th, Shulan International Medical College, Zhejiang Shuren University, Hangzhou, Zhejiang, 310015, China. Email address: 601704@zjsru.edu.cn.

**Chuan Sun**, 1229 Gudun Road, Zhejiang Key Laboratory of Geriatrics and Geriatrics Institute of Zhejiang Province, Zhejiang Hospital, 310030, Hangzhou, China. Email address: sun_chuan@aliyun.com.

**Contents of supplementary materials**

**Figure S1.** Associations of subgroups of ultra-processed foods intake with total testosterone levels. The ultra-processed foods were classified into 18 groups, as presented in table S1. According to the distributions of energy intake from subgroups of ultra-processed foods intake, the groups of sauces, dressings and gravies, salty-snacks, and breads were categorized into three groups based on tertiles values. Other subgroups were classified into intake and un-intake groups. The linear regression models were adjusted for age, race/ethnic, BMI, session time of venipuncture, six-month time period when the sex hormones examination was performed, and ratio of family income to poverty. Recreational physical activity was adjusted additionally for the participants aged 12-19 years old. ^a^ *P* trend values were calculated by treating median values of each group as continuous variable.

**Figure S2.** Associations of subgroup of ultra-processed foods intake with SHBG (sex hormone–binding globulin) levels. The ultra-processed foods were classified into 18 groups, as presented in table S1. According to the distributions of energy intake from subgroups of ultra-processed foods intake, the groups of sauces, dressings and gravies, salty-snacks, and breads were categorized into three groups based on tertiles values. Other subgroups were classified into intake and un-intake groups. The linear regression models were adjusted for age, race/ethnic, BMI, session time of venipuncture, six-month time period when the sex hormones examination was performed, and ratio of family income to poverty. Recreational physical activity was adjusted additionally for the participants aged 12-19 years old. ^a^ *P* trend values were calculated by treating median values of each group as continuous variable.

**Figure S3.** Associations between subgroups of ultra-processed foods intake and FAI (Free androgen index). FAI was calculated using the formula [(TT × 100)/SHBG]. The ultra-processed foods were classified into 18 groups, as presented in table S1. According to the distributions of energy intake from subgroups of ultra-processed foods intake, the groups of sauces, dressings and gravies, salty-snacks, and breads were categorized into three groups based on tertiles values. Other subgroups were classified into intake and un-intake. The linear regression models were adjusted by age, race/ethnic, BMI, session time of venipuncture, six-month time period when the sex hormones examination was performed, and ratio of family income to poverty. Recreational physical activity was adjusted additionally for the participants aged 12-19 years old. ^a^ *P* trend values were calculated by treating median values of each group as continuous variable.

**Figure S4.** Associations of subgroup of ultra-processed foods intake with TT/E2 ratio and E2 (estradiol). The ultra-processed foods were classified into 18 groups, as presented in table S1. According to the distributions of energy intake from subgroups of ultra-processed foods intake, the groups of sauces, dressings and gravies, salty-snacks, and breads were categorized into three groups based on tertiles values. Other subgroups were classified into intake and un-intake. The linear regression models were adjusted by age, race/ethnic, BMI, session time of venipuncture, six-month time period when the sex hormones examination was performed, and ratio of family income to poverty. Recreational physical activity was adjusted additionally for the participants aged 12-19 years old. ^a^ *P* trend values were calculated by treating median values of each group as continuous variable.

**Table S1**. Weighted distribution of proportion of energy intake from subgroup of ultra-processed foods

**Table S2**. Weighted distribution of the sex hormones levels among participants aged 6-19 years in NHANES 2013-2016.

**Table S3.** Associations between percentage of total energy intake from ultra-processed foods and sex hormones levels among participants aged 6-19 years in NHANES 2013-2016 in crude model.

**Table S4.** Associations between percentage of total energy intake from ultra-processed foods and sex hormones levels among participants aged 6-19 years old grouped by puberty status in NHANES 2013-2016 in crude model.

**Figure S1.** Associations of subgroups of ultra-processed foods intake with total testosterone levels. The ultra-processed foods were classified into 18 groups, as presented in table S1. According to the distributions of energy intake from subgroups of ultra-processed foods intake, the groups of sauces, dressings and gravies, salty-snacks, and breads were categorized into three groups based on tertiles values. Other subgroups were classified into intake and un-intake groups. The linear regression models were adjusted by age, race/ethnic, BMI, session time of venipuncture, six-month time period when the sex hormones examination was performed, and ratio of family income to poverty. Recreational physical activity was adjusted additionally for the participants aged 12-19 years old. ^a^ *P* trend values were calculated by treating median values of each group as continuous variable.

**Figure S2.** Associations of subgroup of ultra-processed foods intake with SHBG (sex hormone–binding globulin) levels. The ultra-processed foods were classified into 18 groups, as presented in table S1. According to the distributions of energy intake from subgroups of ultra-processed foods intake, the groups of sauces, dressings and gravies, salty-snacks, and breads were categorized into three groups based on tertiles values. Other subgroups were classified into intake and un-intake groups. The linear regression models were adjusted for age, race/ethnic, BMI, session time of venipuncture, six-month time period when the sex hormones examination was performed, and ratio of family income to poverty. Recreational physical activity was adjusted additionally for the participants aged 12-19 years old. ^a^ *P* trend values were calculated by treating median values of each group as continuous variable.

**Figure S3.** Associations between subgroups of ultra-processed foods intake and FAI (Free androgen index). FAI was calculated using the formula [(TT × 100)/SHBG]. The ultra-processed foods were classified into 18 groups, as presented in table S1. According to the distributions of energy intake from subgroups of ultra-processed foods intake, the groups of sauces, dressings and gravies, salty-snacks, and breads were categorized into three groups based on tertiles values. Other subgroups were classified into intake and un-intake. The linear regression models were adjusted by age, race/ethnic, BMI, session time of venipuncture, six-month time period when the sex hormones examination was performed, and ratio of family income to poverty. Recreational physical activity was adjusted additionally for the participants aged 12-19 years old. ^a^ *P* trend values were calculated by treating median values of each group as continuous variable.

**Figure S4.** Associations of subgroup of ultra-processed foods intake with TT/E2 ratio and E2 (estradiol). The ultra-processed foods were classified into 18 groups, as presented in table S1. According to the distributions of energy intake from subgroups of ultra-processed foods intake, the groups of sauces, dressings and gravies, salty-snacks, and breads were categorized into three groups based on tertiles values. Other subgroups were classified into intake and un-intake. The linear regression models were adjusted by age, race/ethnic, BMI, session time of venipuncture, six-month time period when the sex hormones examination was performed, and ratio of family income to poverty. Recreational physical activity was adjusted additionally for the participants aged 12-19 years old. ^a^ *P* trend values were calculated by treating median values of each group as continuous variable.

**Table S1**. Weighted distribution of proportion of energy intake from subgroup of ultra-processed foods *

| **Ultra-processed foods** | **Min (%)** | **P25 (%)** | **P33 (%)** | **P50 (%)** | **P75 (%)** | **P90 (%)** | **P95 (%)** | **Max (%)** |
| --- | --- | --- | --- | --- | --- | --- | --- | --- |
| Reconstituted meat or fish products | 0 | 0 | 0 | 2.5 | 7.9 | 14.3 | 20.2 | 75.9 |
| Breads ^1^ | 0 | 0 | 1.5 | 4.1 | 8.5 | 14.4 | 18.5 | 68.1 |
| Cakes, cookies, pies, pancakes and pastries | 0 | 0 | 0 | 0 | 0 | 0 | 6.0 | 59.2 |
| Ice cream, ice pops and frozen yogurts | 0 | 0 | 0 | 0 | 3 | 8.9 | 12.8 | 67.2 |
| Desserts and other sugary products ^2^ | 0 | 0 | 0 | 0 | 0 | 1.9 | 3.9 | 38.3 |
| Sugared breakfast cereals | 0 | 0 | 0 | 0 | 5.7 | 10.1 | 13.2 | 32.4 |
| Salty-snacks (Crackers, chips, popcorn) | 0 | 0 | 0.2 | 3.9 | 8.4 | 14.0 | 18.4 | 82.4 |
| Sweet-snacks | 0 | 0 | 0 | 0 | 4.0 | 8.8 | 13.6 | 82.9 |
| Frozen and shelf-stable plate meals | 0 | 0 | 0 | 0 | 3.3 | 11.7 | 16.5 | 82.6 |
| Pizza | 0 | 0 | 0 | 0 | 9.8 | 21.1 | 31.5 | 97.4 |
| Sandwiches and hamburgers on bun (ready-to-eat/heat) | 0 | 0 | 0 | 0 | 0 | 12.1 | 18.1 | 73.9 |
| French fries and other potato products ^3^ | 0 | 0 | 0 | 0 | 1.2 | 6.9 | 10.4 | 53.4 |
| Instant and canned soups | 0 | 0 | 0 | 0 | 0 | 2.9 | 6.9 | 38.3 |
| Sauces, dressings, gravies | 0 | 0 | 0.3 | 0.9 | 2.8 | 5.5 | 8.4 | 38.9 |
| Sugared milk drinks ^4^ | 0 | 0 | 0 | 0 | 3.9 | 9.5 | 13.8 | 49.1 |
| Soft drinks, carbonated | 0 | 0 | 0 | 0 | 5.4 | 10.6 | 14.7 | 41.9 |
| Other sweetened beverages ^5^ | 0 | 0 | 0 | 0.2 | 4.3 | 9.5 | 13.0 | 80.8 |
| Other ultra-processed foods ^6^ | 0 | 0 | 0 | 0.6 | 2.8 | 5.9 | 7.7 | 28.4 |

^1^ Including all types of bread. Processed bread made of flour, water, salt, leavening agents and possibly walnuts, dried fruits, and other whole foods, were included under this group as well, because of the low consumption.

^2^ Including ready-to-eat and dry-mix desserts such as pudding, sugar-based ingredients such as whipped cream, and sweetened canned fruit and fruit sauce.

^3^ Including hash browns, potato puffs, stuffed potatoes, and onion rings (ready-to-eat/heat).

^4^ Including flavored yogurt sweetened with sugar or with low-calorie sweetener, milk shake, and soymilk.

^5^ Including fruit and fruit-flavored, noncarbonated and other sweetened drinks, including presweetened tea and coffee, energy drinks, sports drinks with no milk added, and nonalcoholic wine.

^6^ Including soy products such as meatless patties and fish sticks; dips, spreads, mustard, and catsup; margarine; sugar substitutes, sweeteners, and all syrups (excluding 100% maple syrup); distilled alcoholic drinks.

* The classification of subgroup of ultra-processed foods was based on previous paper (Steele EM, O'Connor LE, Juul F, Khandpur N, Galastri Baraldi L, Monteiro CA, Parekh N, Herrick KA. Identifying and Estimating Ultraprocessed Food Intake in the US NHANES According to the Nova Classification System of Food Processing. J Nutr. 2023 Jan;153(1):225-241. doi: 10.1016/j.tjnut.2022.09.001. Epub 2022 Dec 15. PMID: 36913457; PMCID: PMC10127522).

**Table S2.** Weighted distribution of the sex hormones levels among participants aged 6-19 years in NHANES 2013-2016.

|  | TT  (ng/dL) | E2 (pg/mL) | SHBG (nmol/L) | FAI ^a^ | TT/E2 ^a^ |
| --- | --- | --- | --- | --- | --- |
| **Male children** |  |  |  |  |  |
| GM | 4.4 (0.3) | 2.3 (0.03) | 90.8 (3.1) | 5.1 (0.4) | 1.9 (0.1) |
| Mean | 16.5 (2.2) | 2.5 (0.1) | 103.7 (3.1) | 32.2 (5.4) | 4.3 (0.3) |
| P25 | 2.1 | 2.1 | 66.2 | 1.8 | 1.0 |
| P50 | 3.7 | 2.1 | 99.2 | 3.8 | 1.7 |
| P75 | 6.4 | 2.1 | 137.8 | 10.2 | 2.9 |
| P95 | 84.0 | 4.2 | 183.3 | 112.9 | 21.4 |
| Above LOD (%) | 97.4 | 8.7 | 100 | - | - |
| **Male adolescents** |  |  |  |  |  |
| GM | 280.1 (10.1) | 15.1 (0.5) | 34.7 (1.0) | 804.3 (44.0) | 18.7 (0.4) |
| Mean | 372.3 (8.1) | 18.6 (0.5) | 40.6 (1.4) | 1202.0 (34.3) | 21.4 (0.5) |
| P25 | 224.2 | 11.5 | 23.5 | 665.8 | 14.4 |
| P50 | 372.0 | 18.3 | 34.4 | 1200.6 | 20.0 |
| P75 | 502.9 | 25.1 | 50.0 | 1678.7 | 26.7 |
| P95 | 705.5 | 35.1 | 93.8 | 2459.8 | 38.3 |
| Above LOD (%) | 100 | 93.5 | 100 | - | - |
| **Female children** |  |  |  |  |  |
| GM | 5.4 (0.2) | 5.3 (0.3) | 75.9 (2.8) | 7.1 (0.4) | 1.0 (0.04) |
| Mean | 7.9 (0.3) | 14.0 (1.2) | 88.8 (2.6) | 15.3 (1.3) | 1.4 (0.1) |
| P25 | 2.9 | 2.1 | 53.0 | 2.7 | 0.6 |
| P50 | 5.1 | 2.1 | 84.3 | 6.4 | 1.1 |
| P75 | 9.5 | 13.3 | 117.7 | 19.5 | 1.8 |
| P95 | 22.5 | 57.3 | 171.1 | 57.5 | 3.3 |
| Above LOD (%) | 99.5 | 48.5 | 100 | - | - |
| **Female adolescents** |  |  |  |  |  |
| GM | 23.8 (0.6) | 49.0 (1.9) | 53.8 (1.5) | 44.2 (1.4) | 0.5 (0.02) |
| Mean | 26.6 (0.6) | 80.0 (3.4) | 67.7 (2.5) | 59.3 (2.2) | 0.9 (0.1) |
| P25 | 17.7 | 27.7 | 35.0 | 27.7 | 0.2 |
| P50 | 24.5 | 50.7 | 53.0 | 45.6 | 0.5 |
| P75 | 32.7 | 102.8 | 81.2 | 73.6 | 0.8 |
| P95 | 50.2 | 238.4 | 175.4 | 155.9 | 2.4 |
| Above LOD (%) | 100 | 98.0 | 100 | - | - |

Abbreviation: NHANES, National Health and Nutrition Examination Survey; TT, total testosterone; SHBG, sex hormone–binding globulin; E2, estradiol; FAI, free androgen index; GM, geometric mean; LOD: limit of detection.

^a^ We calculated the FAI using the formula [(TT × 100)/SHBG] and the ratios of TT and E2 (TT/E2) to indirectly assess the approximate amount of circulating free testosterone.

**Table S3.** Associations between percentage of total energy intake from ultra-processed foods and sex hormones levels among participants aged 6-19 years in NHANES 2013-2016 in crude model.

| Percentage of total energy intake from UPFs | TT (%) ^a^  β (95%CI) | SHBG (%) ^a^  β (95%CI) | FAI  β (95%CI) | TT/E2  β (95%CI) | E2 (%) ^a^  β (95%CI) |
| --- | --- | --- | --- | --- | --- |
| **Male children** |  |  |  |  |  |
| Q1 (<55.7%) | Reference | Reference | Reference | - | - |
| Q2 (55.7%-<67.3%) | 6.3 (-26.8, 54.5) | 11.3 (-2.3, 26.7) | -5.8 (-42.4, 30.8) | - | - |
| Q3 (67.3%-<78.1%) | 15.3 (-12.8, 52.3) | 8.3 (-7.2, 26.3) | -14.1 (-41.2, 13.0) | - | - |
| Q4 (≥78.1%) | 19.7 (-8.9, 57.2) | 13.8 (-3.3, 33.9) | -3.2 (-40.6, 34.3) | - | - |
| *P* trend | 0.148 | 0.186 | 0.698 | - | - |
| **Male adolescent** |  |  |  |  |  |
| Q1 (<55.7%) | Reference | Reference | Reference | Reference | Reference |
| Q2 (55.7%-<67.3%) | -4.0 (-23.7, 20.8) | -1.2 (-14.4, 14.1) | **126.2 (20.5, 232.0)** | 0.3 (-2.4, 3.0) | -5 (-21.4, 14.9) |
| Q3 (67.3%-<78.1%) | 7.3 (-12.2, 31.1) | -7.8 (-20.3, 6.7) | **168.0 (21.5, 314.6)** | -0.6 (-2.8, 1.7) | 8.7 (-8.4, 28.9) |
| Q4 (≥78.1%) | 1.6 (-17.3, 24.8) | 5.9 (-6.9, 20.4) | 96.8 (-35.4, 229.0) | 1.1 (-0.5, 2.6) | -3.6 (-19.3, 15.0) |
| *P* trend | 0.663 | 0.571 | 0.186 | 0.329 | 0.987 |
| **Female children** |  |  |  |  |  |
| Q1 (<55.7%) | Reference | Reference | Reference | - | - |
| Q2 (55.7%-<67.3%) | -8.9 (-29.5, 17.6) | 9.3 (-5.3, 26.1) | -1.5 (-6.5, 3.6) | - | - |
| Q3 (67.3%-<78.1%) | 2.2 (-15.8, 24.2) | 9.6 (-9.0, 32.0) | -2.0 (-7.6, 3.6) | - | - |
| Q4 (≥78.1%) | -10.5 (-26.2, 8.6) | 12.0 (-1.1, 26.9) | -1.2 (-6.3, 3.9) | - | - |
| *P* trend | 0.509 | 0.109 | 0.592 | - | - |
| **Female adolescent** |  |  |  |  |  |
| Q1 (<55.7%) | Reference | Reference | Reference | Reference | Reference |
| Q2 (55.7%-<67.3%) | 1.4 (-8.4, 12.2) | -10.0 (-23.3, 5.6) | 3.2 (-3.3, 9.8) | 0.3 (-0.2, 0.9) | -4.0 (-21.9, 18.1) |
| Q3 (67.3%-<78.1%) | -4.9 (-14.3, 5.6) | **-14.0 (-25.0, -1.4)** | 7.6 (-0.7, 16.0) | 0.1 (-0.3, 0.5) | -10.0 (-28.2, 12.7) |
| Q4 (≥78.1%) | 6.7 (-3.1, 17.4) | -8.7 (-22.3, 7.2) | **9.5 (1.5, 17.6)** | -0.1 (-0.4, 0.2) | 5.1 (-13.6, 27.9) |
| *P* trend | 0.429 | 0.208 | **0.023** | 0.247 | 0.842 |

Abbreviation: TT, total testosterone; SHBG, sex hormone–binding globulin; E2, estradiol; FAI, free androgen index; CI, confidence interval; Q1-Q4 represent the quartile values of percent of total energy intake from ultra-processed food;

^a^ Due to TT, SHBG and E2 levels were ln-transformed, the parameter estimates represent percent change in outcome variable with respect to one-unit change of UPFs intake.

**Table S4.** Associations between percentage of total energy intake from ultra-processed foods and sex hormones levels among participants aged 6-19 years old grouped by puberty status in NHANES 2013-2016 in crude model.

| Percentage of total energy intake from UPFs | TT (%) ^a^  β (95%CI) | SHBG (%) ^a^  β (95%CI) | FAI  β (95%CI) | TT/E2  β (95%CI) | E2 (%) ^a^  β (95%CI) |
| --- | --- | --- | --- | --- | --- |
| **Male prepubertal** |  |  |  |  |  |
| Q1 (<55.7%) | Reference | Reference | Reference | - | - |
| Q2 (55.7%-<67.3%) | -6.8 (-28.5, 21.4) | 10.5 (-4.7, 28.3) | 1.0 (-3.3, 5.3) | - | - |
| Q3 (67.3%-<78.1%) | 6.6 (-17.4, 37.7) | 7.9 (-9.3, 28.4) | 1.7 (-3.3, 6.8) | - | - |
| Q4 (≥78.1%) | 19.9 (-7.3, 55.0) | 7.0 (-9.9, 27.0) | 4.2 (-2.8, 11.1) | - | - |
| *P* trend | 0.123 | 0.529 | 0.240 | - | - |
| **Male pubertal** |  |  |  |  |  |
| Q1 (<55.7%) | Reference | Reference | Reference | Reference | Reference |
| Q2 (55.7%-<67.3%) | 2.1 (-7.6, 12.8) | 5.7 (-10.3, 24.4) | 99.5 (-49.6, 248.6) | 1.0 (-1.6, 3.5) | -2.5 (-13.3, 9.7) |
| Q3 (67.3%-<78.1%) | 0.3 (-11.1, 13.2) | 5.1 (-13.5, 27.7) | 74.6 (-68.4, 217.6) | -0.9 (-3.1, 1.2) | 3.9 (-7.5, 16.7) |
| Q4 (≥78.1%) | 4.9 (-6.5, 17.8) | 12.4 (-2.2, 29.1) | -1.3 (-155.5, 152.9) | 1.2 (-0.8, 3.2) | -2.3 (-14.0, 11.0) |
| *P* trend | 0.462 | 0.313 | 0.895 | 0.575 | 0.946 |
| **Female prepubertal** |  |  |  |  |  |
| Q1 (<55.7%) | Reference | Reference | Reference | - | - |
| Q2 (55.7%-<67.3%) | -14.4 (-33.4, 10.1) | -2.5 (-12.7, 8.8) | -1.4 (-5.4, 2.6) | - | - |
| Q3 (67.3%-<78.1%) | -2.9 (-18.7, 16.1) | -6.3 (-17.0, 5.7) | -1.2 (-5.1, 2.7) | - | - |
| Q4 (≥78.1%) | -10.4 (-28.0, 11.4) | 7.1 (-3.6, 19.0) | -1.3 (-4.6, 2.1) | - | - |
| *P* trend | 0.519 | 0.137 | 0.478 | - | - |
| **Female pubertal** |  |  |  |  |  |
| Q1 (<55.7%) | Reference | Reference | Reference | Reference | Reference |
| Q2 (55.7%-<67.3%) | 5.4 (-3.9, 15.5) | -7.7 (-21.2, 8.0) | 8.2 (0.1, 16.4) | 0.3 (-0.2, 0.8) | -0.3 (-17.0, 19.6) |
| Q3 (67.3%-<78.1%) | -1.3 (-11.3, 9.8) | -11.0 (-21.6, 1.1) | 7.3 (-0.8, 15.4) | 0.1 (-0.2, 0.5) | -7.1 (-25.1, 15.2) |
| Q4 (≥78.1%) | 5 (-4.6, 15.6) | -6.8 (-20.5, 9.2) | 9.6 (0.9, 18.3) | -0.1 (-0.4, 0.1) | 6.0 (-10.3, 25.1) |
| *P* trend | 0.614 | 0.301 | **0.051** | 0.229 | 0.770 |

Abbreviation: TT, total testosterone; SHBG, sex hormone–binding globulin; E2, estradiol; FAI, free androgen index; CI, confidence interval; Q1-Q4 represent the quartile values of percent of total energy intake from ultra-processed food;

^a^ Due to TT, SHBG and E2 levels were ln-transformed, the parameter estimates represent percent change in outcome variable with respect to one-unit change of UPFs intake.
